# Top-Down Proteomics of Human Saliva Discloses Significant Variations of the Protein Profile in Patients with Mastocytosis

**DOI:** 10.1021/acs.jproteome.0c00207

**Published:** 2020-06-24

**Authors:** Simone Serrao, Davide Firinu, Alessandra Olianas, Margherita Deidda, Cristina Contini, Federica Iavarone, M. Teresa Sanna, Mozhgan Boroumand, Francisco Amado, Massimo Castagnola, Irene Messana, Stefano Del Giacco, Barbara Manconi, Tiziana Cabras

**Affiliations:** † Dipartimento di Scienze della Vita e dell’Ambiente, Università di Cagliari, 09124 Cagliari, Italy; ‡ Dipartimento di Scienze Mediche e Sanità Pubblica, Università di Cagliari, 09124 Cagliari, Italy; § Dipartimento di Scienze Biotecnologiche di Base, Cliniche Intensivologiche e Perioperatorie, Università Cattolica del Sacro Cuore, 00168 Roma, Italy; ∥ Fondazione Policlinico Universitario A. Gemelli IRCCS, 00168 Roma, Italy; ⊥ Laboratorio di Proteomica e Metabonomica-IRCCS Fondazione Santa Lucia, 100168 Roma, Italy; # QOPNA, Mass spectrometry center, Department of Chemistry, University of Aveiro, 3810-193 Aveiro, Portugal; ∇ Istituto di Scienze e Tecnologie Chimiche “Giulio Natta”, Consiglio Nazionale delle Ricerche, 00185 Roma, Italy

**Keywords:** human saliva, mastocytosis, top-down proteomics

## Abstract

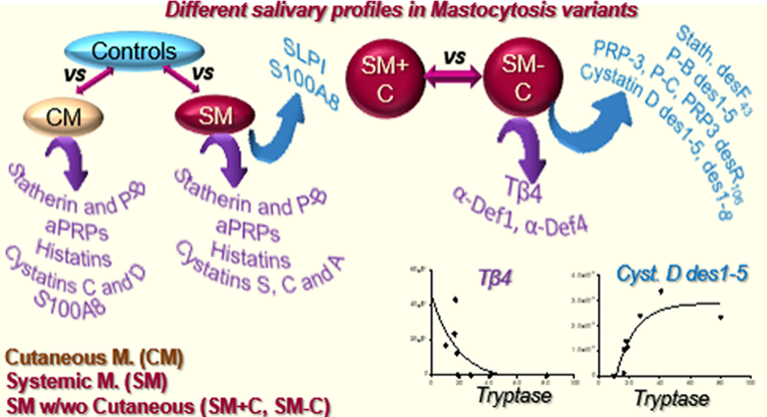

Mastocytosis is a myeloproliferative neoplasm causing abnormal clonal mast cell accumulation in different tissues, such as skin and bone marrow. A cutaneous subtype (CM) is distinguished from a systemic one (SM); SM patients can be grouped into SM with (SM+C) or without (SM-C) additional cutaneous lesions, and their classification is often challenging. This study was purposed to highlight variations in the salivary proteome of patients with different mastocytosis subtypes and compared to healthy controls. A top-down proteomics approach coupled to a label-free quantitation revealed salivary profiles in patients different from those of controls and a down-regulation of peptides/proteins involved in the mouth homeostasis and defense, such as statherin, histatins, and acidic proline-rich proteins (aPRPs), and in innate immunity and inflammation, such as the cathepsin inhibitors, suggesting a systemic condition associated with an exacerbated inflammatory state. The up-regulation of antileukoproteinase and S100A8 suggested a protective role against the disease status. The two SM forms were distinguished by the lower levels of truncated forms of aPRPs, statherin, P-B peptide, and cystatin D and the higher levels of thymosin β4 and α-defensins 1 and 4 in SM-C patients with respect to SM+C. Data are available via ProteomeXchange with identifier PXD017759.

## Introduction

Mastocytosis is an anomalous clonal proliferation and accumulation of mast cells in various tissues^1^ with an estimate prevalence of 1 case *per* 10,000 people.^2^ It can manifest several clinical features, including flushing, pruritus, abdominal pain, diarrhea, hypotension, syncope, and musculoskeletal pain.^3^ Mastocytosis comprises a heterogeneous group of disorders that the World Health Organization (WHO) classifies into subtypes: cutaneous mastocytosis (CM), systemic mastocytosis (SM), and localized mast cells tumors.^4,5^ CM is usually diagnosed in childhood and has a good prognosis, and in many cases, the skin lesion disappears spontaneously during puberty, while CM in adult is a rare condition.^1,6^ WHO divides CM into maculopapular CM, also termed urticaria pigmentosa, diffuse cutaneous mastocytosis, and localized mastocytoma of skin. On the contrary, SM usually develops in adults and it is characterized by infiltration of mast cells in various internal organs, such as bone marrow and the gastrointestinal tract.^1,4^ SM usually occurs as a chronic and indolent disease, also called “indolent systemic mastocytosis”. In some patients, it is possible to diagnose more advanced SM types, such as aggressive SM, SM associated with hematologic neoplasm, and mast cell leukemia.^3,7^ An essential role in the onset of mastocytosis seems to be played by KIT,^8^ the tyrosine kinase receptor expressed on mast cell membranes that regulates the survival of mast cells by interacting with the stem cell factor. Activating mutations in the *c-kit* gene have been revealed in bone marrow, skin, and blood cells of mastocytosis patients. The most common mutation (D816V) involves the catalytic domain of KIT enhancing mast cell proliferation and survival.^9,10^ After activation and degranulation, mast cells generate and release a wide range of allergic and inflammatory mediators, such as histamine, interleukin-3 and 16, tissue necrosis factor, prostaglandin D2, and leukotriene C4, that cause local and distal inflammation.^1,3^ Moreover, mast cells release abundant number of proteolytic enzymes from their secretory granules, especially α-tryptase in both mature and immature pro-tryptase forms. α/β-Tryptases are serine-proteases that preferentially cleaves at R and K residues of small proteins and peptides and also larger substrates such as fibronectin. Even if their biological role was not clarified, tryptases are involved in several mast cell-mediated allergic and inflammatory diseases.^11^ The increased levels of histamine and tryptase are diagnostic tools to diagnose mastocytosis.^12,13^ According to the WHO criteria, the diagnosis is based on laboratory assessments, in particular the tryptase level measuring, clinical manifestations, histopathological skin analyses, or genetic analyses after bone marrow biopsies.^4,5^ Despite the efforts from the WHO in providing more specific and accurate criteria in clinical practical, the diagnosis of the different subtypes of mastocytosis is still difficult. For instance, sometimes patients manifesting the systemic subtype often accumulate mast cells in skin and suffer from additional cutaneous symptoms resembling CM.^14^ The challenging classification of different mastocytosis subtypes drives for the detection of discriminant biomarkers. Saliva is a bodily fluid suitable for the detection of biomarkers because its collection is safe, noninvasive, and painless.^15^ Furthermore, human saliva includes both specific proteins of the oral cavity and proteins common to other tissues and bodily fluids. For this reason, the interest in its prognostic and diagnostic employment is increasing.^16−19^ The use of saliva for the detection of discriminant mastocytosis biomarkers is further attractive in consideration of the mast cell recruitment in allergic, immune, and/or inflammatory reactions and in regenerative processes of the oral cavity,^20^ such as in the pathogenesis of the most common oral lesions.^21^ With these premises, we have investigated the salivary protein profiles of patients with different mastocytosis subtypes and compared them with those of sex/age-matched healthy controls, to evidence significant variations useful for diagnostic purposes. To reach this aim, we applied a top-down proteomic platform, based on high-performance liquid chromatography separation coupled to electrospray-ion trap mass spectrometry (HPLC-ESI-IT-MS) analysis of the acidic soluble intact proteome of human saliva, which was standardized in our previous studies for detecting and quantifying hundreds salivary peptides/proteins.^22−26^ By this approach, it is possible to obtain a profile of the naturally occurring salivary proteome, including isoforms and post-translational modifications (PTMs), and to quantify proteins by a label-free method.

## Materials and Methods

### Reagents and Instrument

All the chemicals and reagents used for high-performance liquid chromatography separation coupled to electrospray-ion trap mass spectrometry (HPLC-ESI-IT-MS) analysis were purchased from Sigma Aldrich (St. Louis, MO, USA). HPLC-ESI-MS analyses were performed with a Surveyor HPLC system connected to an LCQ Advantage mass spectrometer (Thermo Fisher Scientific San Jose, CA, USA). The chromatographic column was a Vydac C8 reverse phase (Hesperia, CA, USA) (150 × 2.1 mm, particle diameter 5 μm). HPLC-high-resolution ESI-MS and MS/MS experiments were carried out using an Ultimate 3000 Micro HPLC apparatus (Dionex, Sunnyvale, CA, USA) equipped with a FLM-3000-Flow manager module and coupled to an LTQ-Orbitrap Elite apparatus (Thermo Fisher). The column was a Zorbax 300SB-C8 (3.5 μm particle diameter; 1.0 × 150 mm).

### Study Subjects and Controls and Clinical Data

Forty-one patients were enrolled from the Internal Medicine and Immunology outpatient clinic of the University of Cagliari. Forty-eight sex/age-matched healthy controls (Ctrl) were enrolled as volunteers among the staff of the Department of Life and Environmental Science, University of Cagliari. The informed consent process agreed with the latest stipulations established by the Declaration of Helsinki. The local review boards authorized the study, and in view of its observational nature, a formal ethical committee approval was obtained. Demographic and clinical features of the included patients are reported in Table 1. The patients have been classified in cutaneous mastocytosis (CM) and systemic mastocytosis (SM) based on the clinical criteria established by the WHO. ^4,5^ A CM group included six patients (four females, mean age ± SD: 37.3 ± 15.7), the SM group included 35 patients (19 females, mean age ± SD: 48 ± 16), among the 35 SM patients, subjects #2, #5, #6, #18, #21, #23, #43, and #46 manifested just systemic symptoms (SM-C), whereas the remaining 27 showed both cutaneous and systemic symptoms (SM + C). Patients who showed hematologic diseases associated with mastocytosis were not enrolled. Serum tryptase levels, using an immune enzymatic method (ImmunoCAP; Thermo Fisher, Waltham, Mass), were measured from blood samples taken at the time of saliva sampling. CM patients showed low levels of tryptase and no mutation in the *c-Kit* gene and in peripheral blood or bone marrow samples. All the SM patients showed high levels of tryptase. Sixteen SM patients showed the mutation of the *c-Kit* gene, mainly D816V. Only patient #9 exhibited M541L mutation, while patient #12 displayed both D816V and M541L mutations. As reported in Table 1, 36.6% of the patients manifested oral aphtosis, of which there are 2 CM, 1 SM-C, and 12 SM + C, and 80.5% of the patients had gastrointestinal (GI) symptoms, of which there are 2 CM, 27 SM + C, and 4 SM-C.

**Table 1:** Age/Gender, Diagnosis, Presence (Y) or Absence (N) of Aphtosis and/or Gastrointestinal (GI) Symptoms, Tryptase Serum Level, and Eventual Mutation of *c-Kit* Genes of each Patient at the Time of the Study

patient	age/gender	diagnosis	secondary cutaneous symptoms	aphtosis	GI	tryptase (μg/L)	mutation(s)
#2	59/F	SM		N	Y	17.5	
#5	54/F	SM		N	Y	16.5	
#6	72/F	SM		N	Y	41.0	
#9	45/M	SM	Y	N	Y	33.6	M541L
#10	44/F	SM	Y	Y	Y	16.2	
#11	58/F	SM	Y	Y	Y	20.2	D816V
#12	53/M	SM	Y	N	Y	5.5	D816V/M541L
#13	35/F	SM	Y	Y	Y	44.7	
#14	22/F	CM		N	N	5.0	
#15	38/F	SM	Y	Y	Y	7.7	D816V
#16	52/M	SM	Y	N	Y	37.5	D816V
#17	46/F	CM		Y	Y	4.5	
#18	55/M	SM		Y	Y	27.1	D816V
#19	48/M	SM	Y	N	Y	16.2	
#20	32/M	SM	Y	N	Y	53.5	D816V
#21	44/F	SM		N	N	16.0	
#22	60/F	SM	Y	N	Y	23.6	
#23	62/F	SM		N	N	80.4	D816V
#24	49/M	SM	Y	N	Y	29.1	D816V
#25	49/F	SM	Y	N	Y	37.4	
#26	35/F	SM	Y	Y	Y	16.5	
#27	67/F	CM		N	N	7.2	
#28	38/F	SM	Y	Y	Y	26.2	D816V
#29	57/M	SM	Y	Y	Y	77.4	D816V
#30	32/M	SM	Y	Y	Y	40.8	D816V
#31	39/M	CM		Y	Y	8.8	
#35	34/M	SM	Y	Y	Y	28.7	
#36	52/F	SM	Y	N	Y	16.1	D816V
#37	28/F	CM		N	N	7.7	
#43	71/F	SM		N	N	9.7	
#45	23/M	CM		N	N	4.3	
#46	75/F	SM		N	N	18.2	
#47	46/F	SM	Y	Y	Y	7.3	
#51	29/M	SM	Y	N	Y	64.5	
#52	44/M	SM	Y	N	Y	7.41	D816V
#58	38/M	SM	Y	N	Y	26.3	
#59	67/F	SM	Y	Y	Y	30.6	
#60	49/F	SM	Y	N	Y	15.8	D816V
#63	56/M	SM	Y	N	Y	60.8	D816V
#64	24/M	SM	Y	Y	Y	11.4	D816V
#66	36/F	SM	Y	N	Y	6.6	D816V

### Sample Collection and Treatment

All the samples of unstimulated whole saliva were collected during the morning (between 10:00 AM and 12:00 PM). Donors, in fasting conditions, were invited to sit assuming a relaxed position and to swallow. Whole saliva was collected as it flowed into the anterior floor of the mouth with a soft plastic aspirator for less than 1 min, transferred to a plastic tube, and cooled on ice. Salivary samples were immediately diluted in a 1:1 v/v ratio with a 0.2% solution of trifluoroacetic acid (TFA) containing 50 μM leu-enkephalin as an internal standard. Then, each sample was centrifuged at 20,000*g* for 15 min at 4 °C. Finally, the supernatant was separated from the precipitate and immediately analyzed by RP-HPLC-ESI-MS or stored at −80 °C until the analysis for up to 2 weeks.

### RP-HPLC-Low-Resolution ESI-MS Analysis

Thirty microliters of acidic extracts was injected in HPLC-low-resolution ESI-MS. The chromatographic eluents were as follows: A, 0.056% TFA in water; and eluent B (acetonitrile/water 80:20 with 0.05% TFA). The gradient applied was linear from 0 to 55% B in 40 min, and from 55 to 100% B in 10 min, at a flow rate of 0.1 mL/min. All the eluate was directed into the ESI source. During the first 5 min of the analysis, the flow was not directed to the mass spectrometer to avoid instrument damages from the high salt concentration. Mass spectra were collected every 3 ms in the *m/z* range 300–2000 in positive ion mode with a resolution of 6000; the MS spray voltage was 5.0 kV, and the capillary temperature was 255 °C. The total ion current (TIC) chromatographic profiles were analyzed to selectively search and quantify the peptides/proteins reported in Table 2, which shows UniProt-KB codes, elution times, and experimental and theoretical average mass values (Mav) of the proteins/peptides, included in the study. Table 2 also reports the multiply charged ions used for the extracted ion current (XIC) search that were selected excluding values common to other closely eluting proteins. A window of ±0.5 Da was used to extract XIC peaks. Experimental Mav values were obtained by deconvolution of averaged ESI-MS spectra automatically performed by using MagTran 1.0 software.^27^ Mav and elution times of proteins/peptides were compared with those determined on salivary samples, under the same experimental conditions, in our previous studies.^22^ Experimental Mav values were also compared with the theoretical ones available at the UniProt-KB human databank (http://us.expasy.org/tools). XIC peak areas were integrated by using the following peak parameters: baseline window, 15; area noise factor, 50; peak noise factor, 50; peak height, 15%; and tailing factor, 1.5. The area of the XIC peaks is proportional to the protein concentration, and, under constant analytical conditions, it allows performing relative quantification of the same protein in different samples.^23,28,29^ The estimated percentage error of the XIC procedure was <8%. Eventual dilution errors occurring during sample collection were corrected by normalizing XIC peak areas of peptides/proteins with the XIC peak area of leu-enkephalin used as the internal standard, as described in a previous study.^30^

**Table 2:** UniProt-KB Code, Experimental and Theoretical Average Mass Values ± Standard Deviations (SD), Average (Mav) and Monoisotopic ([M + H]^+^), Elution Times of Proteins and Peptides Analyzed, and Their PTMs^b^

proteins/peptides	el. time (min ± 0.5)	Mav ± SD exper. (theor)	*m/z* (charge) for XIC search	[M + H]^+^ ± SD exper. (theor)	PTMs	*m/z* (charge) for MS/MS
Acid proline-rich proteins (PRP-1 and PRP-3 types)^a^						
PRP-1 (P02810)	22.2	15515 ± 2 (15514–15,515)	1293.9(+12), 1194.4(+13), 1035.3(+15), 970.7(+16), 913.6(+17)	15506.1 ± 0.1 (15506.24–15507.22)	Q1 pyroglutamination, S8 and S22 phosphorylation	1293.68(+12); 1194(+13); 1035.28 (+15); 970.58 (+16); 862.85(+18)
PRP-1 1P	22.9	15435 ± 2 (15434–15,435)	1287.2(+12), 1188.3(+13), 1030.0(+15), 965.7(+16), 908.9(+17)	15426.1 ± 0.1 (15426.27–15427.26)	Q1 pyroglutamination, S8 or S22 phosphorylation	1287.01(+12); 1188.3(+13); 1030.0 (+15)
PRP-1 0P	23.2	15355 ± 2 (15354–15,355)	1280.5(+12), 1182.1(+13), 1024.6(+15), 960.7(+16), 904.2(+17)	15346.1 ± 0.1 (15346.31–15347.29)	Q1 pyroglutamination	1534.42 (+10); 904.08 (+17)
PRP-1 3P	21.6	15595 ± 2 (15594–15,595)	1418.7(+11), 1300.5(+12), 1200.6(+13), 1040.6(+15), 975.7(+16)	15585.8 ± 0.1 (15586.21–15587.19)	Q1 pyroglutamination, S8, S17, and S22 phosphorylation	1733.70 (+9); 1560.08 (+10); 1114.61 (+14); 975.56 (+16)
PRP-3 (P02810)	22.8	11161 ± 1 (11161–11,162)	1595.5(+7), 1396.2(+8), 1015.7(+11), 931.1(+12), 859.6(+13)	11155.01 ± 0.07 (11156.08–11157.06)	Q1 pyroglutamination, S8 and S22 phosphorylation	931.09(+12); 798.07(+14); 745.00(+15)
PRP-3 1P	23.4	11081 ± 1 (11081–11,082)	1584.1(+7), 1386.2(+8), 1008.4(+11), 924.5(+12), 853.4(+13)	11075.98 ± 0.07 (11076.11–11077.09)	Q1 pyroglutamination, S8 or S22 monophosphorylation	1386.13(+8); 1008.28(+11); 853.31(+13)
PRP-3 0P	23.8	11001 ± 1 (11001–11,002)	1376.2(+8), 1101.2(+10), 917.8(+12) 786.8(+14)	10996.01 ± 0.07 (10996.14–10997.13)	Q1 pyroglutamination	1001.10(+11); 786.72(+14)
PRP-3 desR_6_	22.8	11004 ± 1 (11005–11,006)	1573.2(+7), 1223.8(+9), 1001.5(+11), 847.6(+13)	10999.85 ± 0.07 (10999.97–11000.96)	C-term. R106 removal from PRP-3	1001.45(+11); 918.08(+12)
P-C peptide (P02810)	15.0	4370.9 ± 0.4 (4370.8)	1457.9(+3), 1093.7(+4)	4369.19 ± 0.03 (4369.20)		1457.07(+3); 1093.05(+4); 874.64(+5); 729.04(+6)
Statherin						
statherin (P02808)	29.2	5380.0 ± 0.5 (5379.7)	1794.2(+3), 1345.9(+4), 1076.9(+5)	5377.46 ± 0.03 (5377.45)	S2 and S3 phosphorylation	1793.17(+3); 1345.12(+4); 1076.30(+5)
statherin 1P	28.9	5299.9 ± 0.5 (5299.7)	1767.6(+3), 1325.9(+4), 1060.9(+5)	5297.46 ± 0.03 (5297.48)	S2 or S3 phosphorylation	1325.12(+4); 1060.30(+5)
statherin des-F_43_	27.8	5232.4 ± 0.5 (5232.5)	1745.1(+3), 1309.1(+4), 1047.5(+5)	5230.39 ± 0.03 (5230.38)	C-term. F43 removal from statherin	1744.13(+3); 1308.35(+4); 1046.88(+5)
statherin desT_42_-F_43_	27.9	5131.2 ± 0.5 (5131.4)	1711.4(+3), 1283.8(+4), 1027.2(+5)	5129.34 ± 0.03 (5129.33)	C-term. T42 and F43 removal from statherin	1710.45(+3); 1283.09(+4); 1026.67(+5)
statherin desD_1_	28.7	5264.7 ± 0.5 (5264.6)	1755.9(+3), 1317.2(+4), 1053.9(+5)	5262.43 ± 0.03 (5262.42)	N-term D1 removal from statherin	1754.81(+3); 1316.36(+4); 1053.29(+5)
statherin des1-9	28.5	4127.9 ± 0.4 (4127.6)	1376.9(+3), 1032.9(+4)	4126.00 ± 0.02 (4125.99)	removal of D_1_SSEEKFLR_9_ from statherin	1376.00(+3); 1032.25(+4)
statherin des1-10	28.0	3971.3 ± 0.4 (3971.4)	1986.7(+2), 1324.8(+3)	3969.88 ± 0.02 (3969.89)	Removal of D_1_SSEEKFLRR_10_ from statherin	1323.96(+3); 993.22(+4)
statherin des1-13	27.5	3645.2 ± 0.4 (3645.0)	1823.6(+2), 1216.1(+3)	3643.69 ± 0.02 (3643.68)	Removal of D_1_SSEEKFLRRIGR_13_ from statherin	1215.23(+3); 911.68(+4)
P-B peptide						
P-B peptide (P02814)	30.0	5792.9 ± 0.5 (5792.7)	1932.0(+3), 1449.2(+4), 1159.6(+5)	5790.05 ± 0.04 (5790.04)		1930.68(+3); 1448.27(+4); 1158.82(+5)
P-B des1-4	30.0	5371.0 ± 0.5 (5371.3)	1791.4(+3), 1343.8(+4), 1075.3(+5)	5368.83 ± 0.03 (5368.82)	removal of Q_1_RGP_4_ from P-B	1790.25(+3); 1342.96(+4); 1074.57(+5)
P-B des1-5	30.3	5215.0 ± 0.5 (5215.1)	1739.4(+3), 1304.8(+4), 1044.0(+5)	5212.74 ± 0.03 (5212.73)	removal of Q_1_RGPR_5_ from P-B	1303.93(+4); 1043.35(+5)
P-B des1-7	30.1	5060.1 ± 0.5 (5060.9)	1688.0(+3), 1266.2(+4), 1013.2(+5)	5058.66 ± 0.03 (5058.65)	removal of Q_1_RGPRGP_7_ from P-B	1686.88(+3); 1265.42(+4); 1012.54(+5)
P-B des1-12	27.5	4549.0 ± 0.5 (4549.3)	1517.5(+3), 1138.3(+4)	4547.41 ± 0.02 (4547.41)	removal of Q_1_RGPRGPYPPGP_12_ from P-B	1137.61(+4)
Histatins						
Hst-1 (P15515)	21.9	4928.2 ± 0.5 (4928.2)	1644.1(+3), 1233.5(+4)	4926.19 ± 0.03 (4926.20)	S2 phosphorylation	986.05(+5); 821.87(+6); 704.61(+7)
Hst-1 0P	22.0	4848.2 ± 0.5 (4848.2)	1617.4(+3), 1213.5(+4)	4846.24 ± 0.03 (4846.23)		970.05(+5)
Hst-3 (P15516)	17.7	4062.2 ± 0.4 (4062.4)	1355.1(+3), 1016.6(+4)	4060.97 ± 0.02 (4060.98)		1015.99(+4); 813.00(+5); 677.67(+6)
Hst-3 1/25	14.3	3192.4 ± 0.3 (3192.5)	1065.1(+3), 799.1(+4)	3191.63 ± 0.02 (3191.62)	removal of S_26_NYLYDN_32_ from Hst-3	400.09(+8); 456.95(+9)
Hst-3 1/24	14.6	3036.5 ± 0.3 (3036.3)	1013.2(+3), 760.1(+4)	3035.53 ± 0.02 (3035.52)	removal of S_26_NYLYD_31_ from Hst-3	608.11(+5); 506.93(+6); 434.65(+7)
Cystatins						
cystatin A (P01040)	31.8	11005 ± 2 (11006.5)	1001.59(+11), 1101.59(+10), 1223.94(+9), 1376.81(+8), 1573.36(+7), 1835.42(+6)	11000.65 ± 0.07 (11000.67)		1376.83(+8); 847.74(+13); 787.19(+14); 734.85(+15);
cystatin A Nα-Ac	33	11047 ± 2 (11048.5)	1005.41(+11), 1105.85(+10), 1228.61(+9), 1382.06(+8), 1579.36(+7), 1842.42(+6)	11042.55 ± 0.07 (11042.68)	N-terminal acetylation	1381.79(+8); 789.98(+14)
cystatin B-SSG (P04080)	32.8	11486 ± 2 (11486.9)	1915.5(+6), 1642.0(+7), 1436.9(+8), 1277.3(+9), 1149.7(+10), 1045.3(+11)	11480.61 ± 0.07 (11480.68)	N-terminal acetylation, C3 glutathionylation	1436.72(+8); 1277.31(+9); 766.91(+15), 718.79(+16)
cystatin C (P01034)	35.1	13342 ± 2 (13343.1)	1483.57(+9), 1335.32(+10), 1214.02(+11), 1112.93(+12), 1027.40(+13)	13335.31 ± 0.08 (13335.58)	2 disulfide bridges	1112.81(+12); 890.50(+15)
cystatin C Mox	35.3	13358 ± 1 (13358.4)	1485.28(+9), 1336.85(+10), 1215.41(+11), 1114.21(+12), 1028.58(+13)	13351.60 ± 0.08 (13351.57)	M14 oxidation of cystatin C	1114.23(+12); 954.20(+14)
cystatin D-R_26_ des1-8 (P28325)	38.0	13163 ± 2 (13163.0)	1646.40 (+8), 1463.60 (+9), 1317.30 (+10), 1197.60 (+11), 1097.90 (+12), 1013.50 (+13)	13155.25 ± 0.08 (13155.48)	removal of G_1_SASAQSR_8_, 2 disulfide bridges	941.09(+14); 823.71(+16)
cystatin D-R_26_ des1-5	37.7	13517 ± 2 (13517.3)	1690.70(+8), 1502.90(+9), 1352.70 (+10), 1229.80 (+11), 1127.4 (+12), 1040.40 (+13)	13509.41 ± 0.08 (13509.65)	Q1 pyroglutamination after removal of G_1_SASA_5_, 2 disulfide bridges	1502.63(+9), 1040.87(+13) 966.48(+14); 845.72(+16)
cystatin S (P01036)	35.3	14186 ± 2 (14185)	1774.3(+8), 1577.2(+9), 1419.6(+10), 1290.6(+11), 1183.2(+12), 1092.2(+13), 1014.3(+14)	14176.61 ± 0.08 (14176.81)	2 disulfide bridges	946.58(+15); 835.22(+17)
cystatin S1	35.3	14266 ± 2 (14265)	1784.3(+8), 1586.1(+9), 1427.6(+10), 1297.9(+11), 1189.8(+12), 1098.4(+13), 1020.0(+14)	14256.66 ± 0.09 (14256.77)	S3 phosphorylation of cystatin S, 2 disulfide bridges	1098.37(+13); 1019.69(+14); 951.91(+15)
cystatin S1ox	35.3	14281 ± 2 (14280.7)	1786.40(+8), 1589.70 (+9), 1429.30 (+10), 1299.50 (+11), 1191.30 (+12), 1099.70 (+13)	14272.66 ± 0.09 (14272.77)	W23 oxidation of cystatin S1	1299.08(+11); 1099.36(+13); 894.67(+16)
cystatin S2	35.3	14346 ± 2 (14345)	1794.3(+8), 1595.0(+9), 1435.6(+10), 1305.2(+11), 1196.5(+12), 1104.5(+13), 1025.7(+14)	14336.58 ± 0.09 (14336.74)	S1 and S3 phosphorylation of cystatin S, 2 disulfide bridges	957.31(+15); 897.55(+16)
cystatin SN (P01037)	34.6	14312 ± 2 (14313)	1790.0(+8), 1591.2(+9), 1432.2(+10), 1302.1(+11), 1193.7(+12), 1101.9(+13), 1023.3(+14)	14304.03 ± 0.09 (14304.09)	2 disulfide bridges	1023.29(+14); 796.01(+18); 716.50(+20)
cystatin SNox	34.6	14328 ± 2 (14328)	1792.30(+8), 1593.20 (+9), 1434.00 (+10), 1303.30 (+11), 1195.20 (+12), 1103.30 (+13)	14320.09 ± 0.09 (14319.84)	2 disulfide bridges, W23 oxidation	843.64(+17); 754.26(+19); 717.35(+20); 682.52(+21)
cystatin SA (P09228)	36.8	14347 ± 2 (14346)	1794.4(+8), 1595.1(+9), 1435.7(+10), 1305.3(+11), 1196.6(+12), 1104.6(+13), 1025.8(+14)	14337.87 ± 0.09 (14338.01)	1 disulfide bridge	897.50(+16); 844.88(+17); 798.78(+18);
Antileukoproteinase						
SLPI (P03973)	26.2	11709 ± 1 (11710)	1952.64(+6), 1673.84(+7), 1464.73(+8), 1302.10(+9)	11702.41 ± 0.07 (11702.36)	8 disulfide bridges	1171.82(+10); 1065.47(+11); 976.69(+12); 837.45(+14)
α-Defensins						
α-defensin 1 (P59665)	23.5	3442.5 ± 0.3 (3442.1)	1772.03(+2), 1148.36(+3), 861.52(+4)	3440.45 ± 0.02 (3440.519)	3 disulfide bridges	861.14(+4); 689.11(+5)
α-defensin 2 (P59665/6)	23.5	3370.7 ± 0.3 (3370.9)	1686.49(+2), 1124.66(+3), 843.75(+4)	3370.41 ± 0.02 (3370.484)	3 disulfide bridges	843.61(+4); 675.10(+5)
α-defensin 3 (P59666)	23.5	3485.8 ± 0.3 (3486.1)	1744.03(+2), 1163.03(+3), 872.52(+4)	3484.53 ± 0.02 (3484.509)	3 disulfide bridges	872.11(+4); 698.09(+5)
α-defensin 4 (P12838)	27.2	3709.1 ± 0.3 (3709.4)	1855.71(+2), 1237.48(+3), 928.36(+4)	3707.68 ± 0.02 (3707.767)	3 disulfide bridges	928.18(+4); 742.74(+5);
Thymosin β4						
Tβ4 (P62328)	18.5	4963.7 ± 0.5 (4963.5)	1655.51(+3), 1241.88(+4), 993.71(+5)	4961.47 ± 0.03 (4961.494)	N-terminal acetylation	710.08(+7), 621.32(+8)
S100A proteins						
S100A8 (P05109)	40.4	10834 ± 2 (10834.6)	1355.3(+8), 1204.8(+9), 1084.5(+10), 985.9(+11)	10827.41 ± 0.07 (10828.66)		903.62(+12); 834.11(+13); 774.75(+14)
S100A8 SNO	40.8	10863 ± 2 (10863.5)	1358.9(+8), 1208.1(+9), 1087.3(+10), 988.6(+11)	10857.37 ± 0.07 (10857.65)	C42 nitrosylation	1087.49(+10); 680.03(+16); 640.03(+17)
S100A8 hyperoxidized	39.3	10915 ± 2 (10914.6)	1365.3(+8), 1213.7(+9), 1092.5(+10), 993.2(+11)	10908.40 ± 0.07 (10908.63)	C42-SO_3_H, W54 dioxidation or C42-SO_3_H, W54, and M78 oxidation	993.20(+11); 910.37(+12); 683.16(+16); 642.85(+17)
S100A9(S) (P06702)	42.2	12690 ± 2 (12689.3)	1410.9(+9), 1269.9(+10), 1154.6(+11), 1058.4(+12), 977.1(+13)	12682.21 ± 0.08 (12682.29)	N-term. Acetylation after M_1_TCKM_5_ removal	1410.67(+9); 1269.93(+10); 977.22(+13); 793.95(+16); 747.48(+17); 705.85(+18);
S100A9(S) 1P	42.2	12770 ± 2 (12769.3)	1419.8(+9), 1277.9(+10), 1161.8(+11), 1065.1(+12), 983.3(+13)	12762.05 ± 0.08 (12762.26)	T108 phosphorylation of S100A9(S)	1161.66(+11); 798.96(+16); 710.29(+18)
S100A9(S)ox	41.3	12706 ± 2 (12705.3)	1412.7(+9), 1271.5(+10), 1156.0(+11), 1059.8(+12), 978.3(+13)	12698.14 ± 0.08 (12698.29)	M89, or 78 or 76 or 58 oxidation of S100A9(S)	1271.41(+10); 1155.92(+11); 908.51(+14)
S100A9(S)ox 1P	41.3	12786 ± 2 (12785.3)	1421.9(+9), 1279.5(+10), 1163.3(+11), 1066.4(+12), 984.5(+13)	12778.21 ± 0.08 (12778.25)	T108 phosphorylation of S100A9(S)ox	1419.82(+9); 983.26(+13); 913.10(+14)

a PRP-1 type includes the three isobaric variants PRP-1, PRP-2, and Pif-s; PRP-3 type includes the three isobaric variants PRP-3, PRP-4, and Pif-f.

b Table reported also the *m/z* values and charge of the multiply charged ions selected for XIC search in HPLC-low resolution MS, and those ones used for high-resolution MS/MS characterization.

## Statistical Analysis

GraphPad Prism software (version 6.0) was used to calculate means and standard deviations of the protein XIC peak areas and to perform statistical analysis. Data distributions were tested for normality by the D’Agostino–Pearson test. The comparisons between the three groups of patients and that of the controls and between the three groups of patients have been performed with Mann–Whitney and Welch-corrected *t* tests, depending on the distribution (skewed or normal) and the variance (unequal or homogeneous). Statistical analysis has been considered significant when the *p* value was <0.05. The data were also analyzed by a volcano plot, which plots on the *X* axis the log_2_(fold-change) mean value of XIC peak areas from patient/control ratio or patient/patient ratio and on the *Y* axis the log_10_ of *p* value from the *t*-test analysis. Correlation analyses, between the XIC peak area of the proteins/peptides under analysis and the tryptase levels of each patient, were performed with the Spearman test and considered significant when the *p* value was <0.05.

### RP-HPLC-High-Resolution ESI-MS/MS Analysis

The identification of the peptides and proteins investigated in this study and reported in Table 2 was performed by HPLC-high-resolution ESI-MS/MS (LTQ-Orbitrap Elite) operating in “Intact Protein Mode”, with the delta HCD (higher energy collisional dissociation) vacuum pressure reduced to 0.1.^31^ The chromatographic separation was carried out using eluent A: 0.1% (v/v) aqueous formic acid (FA) and eluent B: 0.1% (v/v) FA in ACN–water 80/20. The gradient was as follows: 0–2 min 5% B, 2–10 min from 5 to 25% B (linear), 10–25 min from 25 to 34% B, 25–45 min from 34 to 70% B, and 45–55 min from 70 to 90% B at a flow rate of 50 μL/min. The injection volume was 19 μL. Full MS experiments were performed in positive ion mode with a mass range from 400 to 2000 *m*/*z* at a resolution of 120,000 (at 400 *m*/*z*). The capillary temperature was 275 °C, the source voltage was 4.0 kV, and the S-Lens RF level was 69%. In data-dependent acquisition mode, the five most abundant ions were acquired and fragmented by using collision-induced dissociation (CID) and higher energy collisional dissociation (HCD) with a 35% normalized collision energy for 10 ms, isolation width of 5 *m*/*z*, and activation *q* of 0.25. HPLC-ESI-MS and MS/MS data were generated by Xcalibur 2.2 SP1.48 (Thermo Fisher Scientific, CA) using default parameters of the Xtract program for the deconvolution. MS/MS data were analyzed by both manual inspection of the MS/MS spectra recorded along the chromatogram and the Proteome Discoverer 2.2 software elaboration based on the SEQUEST HT cluster as a search engine (University of Washington, licensed to Thermo Electron Corporation, San Jose, CA) against the UniProtKB human databank (188,453 entries, release 2019_03). For peptide matching, high-confidence filter settings were applied: the peptide score threshold was 2.3, and the limits were Xcorr scores >1.2 for singly charged ions and 1.9 and 2.3 for doubly and triply charged ions, respectively. The false discovery rate (FDR) was set to 0.01 (strict) and 0.05 (relaxed), and the precursor and fragment mass tolerance were 10 ppm and 0.5 Da, respectively. N-Terminal pyroglutamination of E or Q residues, phosphorylation on S and T residues, N-terminal acetylation, oxidation of M and W residues, glutathionylation, nitrosylation, and sulfonic acid of C residues were selected as dynamic modifications. Because of the difficulties of the automated software to detect with high confidence every protein and their fragments, the structural information derives in part from manual inspections of the MS/MS spectra, obtained by both CID and HCD fragmentation, against the theoretical ones generated by MS-Product software available at the Protein Prospector website (http://prospector.ucsf.edu/prospector/mshome.htm). All the MS/MS spectra were manually verified by utilizing all the fragmentation spectra with an acceptable number of fragment ions. The mass spectrometry proteomics data have been deposited into the ProteomeXchange Consortium (http://www.ebi.ac.uk/pride) via the PRIDE^32^ partner repository with the dataset identifier PXD017759.

## Results

**Figure 1:**
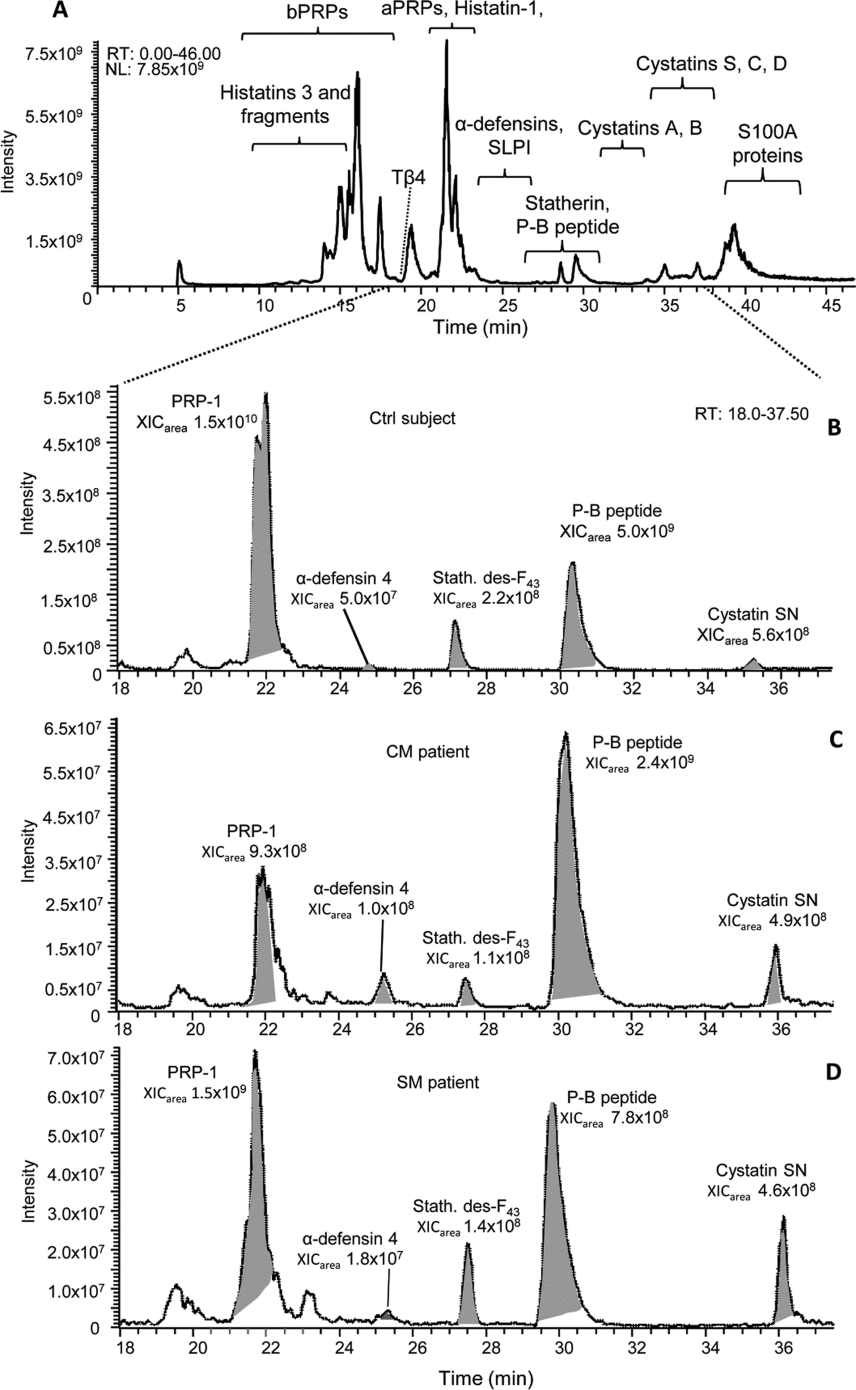
(A) Typical TIC chromatographic profile obtainable by HPLC-ESI-MS conditions used in this study. Enlargement between 18.0 and 37.5 min of the XIC profiles obtained from (B) a Ctrl subject, (C) a CM patient, and (D) an SM patient, by simultaneously searching the *m*/*z* ions specific to some components with different elution times (PRP-1, α-defensin 4, statherin desF43, P-B peptide, and cystatin SN).

To analyze the salivary samples by RP-HPLC-ESI-MS, whole saliva collected from healthy subjects and patients was treated with TFA aqueous solution. This treatment minimizes the activity of the exogenous and endogenous proteases present in saliva, thus preventing the degradation of proteins and peptides;^23^ on the other hand, it causes the precipitation of several high-molecular-weight salivary proteins.^33^ RP-HPLC-ESI-MS top-down analysis of the acid-soluble fraction of human saliva allowed detecting the proteins/peptides, and their PTM derivatives are reported in Table 2, as well as performing a comparative label-free quantification of these components in different sets of samples. The typical TIC chromatographic profile obtainable under our experimental conditions is shown in Figure 1A.

In this figure, the elution ranges of several salivary protein/peptide families are indicated: acidic proline-rich proteins (aPRPs), histatins (Hst-1, Hst-3, Hst-3 1/24, Hst-3 1/25), statherin, P-B peptide, thymosin β4 (Tβ4), cystatins A, B, C, and D (variant R_26_), and S-type, S100A8, S100A9, and antileukoproteinase (SLPI). Many proteoforms already characterized in our previous proteomic studies on human saliva were included in this study.^22−24,34−37^ Protein identifications were confirmed by high-resolution MS/MS and the monoisotopic mass values [M + H]^+1^, and the *m/z* values of the multiply charged ion precursors selected for MS/MS sequencing are reported in Table 2. MS/MS data have been deposited into the ProteomeXchange Consortium.

### Quantitative Comparison between Controls and Patients

Quantitative differences detected between controls and patients were assessed by the XIC procedure. An example of this approach is reported in Figure 1B–D, showing the XIC profiles between 18.0 and 37.5 min obtained by searching the *m/z* ions specific to PRP-1, α-defensin 4, statherin desF_43_, P-B peptide, and cystatin SN in saliva samples from a Ctrl subject, a CM patient, and an SM patient, respectively. The *m/z* ions used to extract the multi-XIC profiles in Figure 1 are reported in Table 2. The statistical comparison between the XIC peak area values of proteins/peptides measured in Ctrl, CM, and SM groups provided the results resumed in Table 3. In the case of cystatins B, C, S1, SN, and S100A8, all the proteoforms of the same protein showed the same trend and the sum of their XIC peak areas is reported in Table 3. All the aPRP proteoforms, except for PRP-3 desR_106_, showed a significant lower abundance in all the patients with respect to the Ctrls. Among the investigated statherin proteoforms, only statherin desT_42_-F_43_ did not show variations between the three groups, whereas the di- and mono-phosphorylated forms and the desF_43_, desD_1_, des1-9, des1-10 and des1-13 truncated forms were significantly less concentrated in the saliva of SM patients than in Ctrls. Statherin des1-13 resulted in a lower concentration also in the CM group than in controls. The same results were obtained for the P-B peptide and its truncated form P-B des1-7. Significant decreased levels of histatins in both SM and CM patients with respect to the controls were observed. Apart from histatin 1, almost all the investigated histatins were undetectable in CM patients (Table 3), being only histatin 3 detected in one CM patient. Cystatin C was detected in 32 out of 48 healthy controls but sporadically in the patients: in 3 out of 35 SM patients and in 1 out of 6 CM patients. In this last case, only the M_14_-oxidized derivative was observed. Thus, for these proteins/peptides, the statistical comparison was not performed. The S-type cystatins, S1, S2, SN, and SA, were exhibited in a significantly lower level in SM patients than in controls (Table 3). The lowest level of cystatin A was found in the SM patients, while CM patients exhibited levels comparable with controls. The variant R_26_ of cystatin D was detected as truncated proteoforms due to either the removal of the first eight N-terminal residues, des1-8, or the removal of the first five residues, des1-5, this latter proteoform carrying a N-terminal pyroglutamination. The two proteoforms of cystatin D-R_26_ were almost absent in CM patients. No variation was observed for cystatin B levels in the patients. S100A8 was sporadically detected as an unmodified proteoform: it was found in 4 SM patients and in 4 Ctrls but never in CM patients. Similarly, the hyperoxidized form of S100A8 was found in just 2 SM patients and in 3 Ctrls, at very low levels, while nitrosylated S100A8 was found at a higher frequency: in 1 CM patient, 8 SM patients, and in 3 Ctrls. Statistical analysis performed by considering the sum of the XIC peak of the different proteoforms of S100A8 highlighted its significantly higher abundance in SM patients with respect to that in controls. Similar results were obtained for antileukoproteinase (SLPI), which has been found in 10 out of 48 healthy controls, whereas the patient groups showed a higher frequency, 18 out of 35 SM patients and 3 out of 6 CM patients (Table 3). The SM group exhibited a significant higher abundance of SLPI than healthy controls: the significant difference was confirmed also by excluding the two outlier points, and indeed the *p* value changed from 0.005 to 0.01.

**Table 3:** XIC Peak Areas Values (Mean ± SD), Frequencies, and *p* Values Obtained by Statistical Analysis by Comparing the Three Patients’ Groups with Respect to the Controls and Each Other^a^

		XIC peak area ×10^8^ (mean ± SD) and frequency			*p* values	
no.	protein/peptide	CM	SM	Ctrls	CM vs Ctrls	SM vs Ctrls
#1	PRP-1	17.8 ± 10.1 (6/6)	27.4 ± 27.2 (35/35)	99.9 ± 70.9 (48/48)	<0.0001 ↓CM	< 0.0001 ↓SM
#2	PRP-1 1P	2.5 ± 3.1 (5/6)	3.7 ± 5.2 (29/35)	12.8 ± 12.9 (47/48)	0.002 ↓CM	< 0.0001 ↓SM
#3	PRP-1 0P	**(0/6)**	0.09 ± 0.3 (6/35)	0.5 ± 0.9 (22/48)	NA	0.003 ↓SM
#4	PRP-1 3P	0.2 ± 0.2 (4/6)	0.4 ± 0.6 (22/35)	2.0 ± 1.9 (44/48)	0.002 ↓CM	< 0.0001 ↓SM
#5	PRP-3	5.9 ± 4.4 (6/6)	9.5 ± 11.3 (33/35)	35.1 ± 29.3 (48/48)	0.0002 ↓CM	< 0.0001 ↓SM
#6	PRP-3 1P	1.1 ± 1.0 (6/6)	1.5 ± 1.8 (29/35)	4.9 ± 4.1 (47/48)	0.002 ↓CM	< 0.0001 ↓SM
#7	PRP-3 0P	**(0/10)**	0.004 ± 0.02 (2/35)	0.4 ± 1.1 (18/48)	NA	0.0003 ↓SM
#8	PRP-3 desR_106_	3.1 ± 2.7 (6/6)	2.8 ± 5.9 (26/35)	3.8 ± 6.2 (40/48)	•	•
#9	P-C peptide	4.1 ± 4.4 (6/6)	7.0 ± 10.0 (33/35)	20.4 ± 16.2 (48/48)	0.001 ↓CM	< 0.0001 ↓SM
#10	statherin	5.4 ± 6.6 (5/6)	6.0 ± 6.6 (34/35)	13.4 ± 10.1 (47/47)	0.02 ↓CM	0.0001 ↓SM
#11	Stath. 1P	0.2 ± 0.2 (3/6)	0.1 ± 0.2 (17/35)	0.3 ± 0.4 (32/47)	•	0.03 ↓SM
#12	Stath. des-F_43_	1.1 ± 1.0 (6/6)	1.0 ± 0.9 (34/35)	1.9 ± 1.8 (46/47)	•	0.006 ↓SM
#13	Stath. desT_42_-F_43_	0.7 ± 0.4 (6/6)	0.4 ± 0.4 (25/35)	0.7 ± 0.7 (46/47)	•	•
#14	Stath. desD_1_	0.7 ± 0.9 (5/6)	0.5 ± 0.5 (31/35)	0.8 ± 0.8 (46/47)	•	0.02 ↓SM
#15	Stath. des1-9	0.1 ± 0.2 (5/6)	0.2 ± 0.4 (18/35)	0.7 ± 0.7 (40/47)	•	< 0.0001 ↓SM
#16	Stath. des1-10	0.2 ± 0.2 (5/6)	0.3 ± 0.4 (24/35)	0.5 ± 0.5 (44/47)	•	0.001 ↓SM
#17	Stath. des1-13	0.1 ± 0.1 (4/6)	0.1 ± 0.2 (22/35)	0.3 ± 0.2 (45/47)	0.04 ↓CM	< 0.0001 ↓SM
#18	P-B peptide	7.6 ± 7.9 (6/6)	9.2 ± 7.8 (34/35)	21.9 ± 14.3 (47/47)	0.004 ↓CM	< 0.0001 ↓SM
#19	P-B des1-4	0.9 ± 0.6 (6/6)	1.0 ± 0.8 (31/35)	1.5 ± 1.7 (39/48)	•	•
#20	P-B des1-5	3.0 ± 3.6 (6/6)	1.8 ± 2.4 (30/35)	2.3 ± 2.8 (42/48)	•	•
#21	P-B des1-7	1.7 ± 0.9 (6/6)	1.7 ± 1.5 (32/35)	4.4 ± 2.8 (47/48)	0.007 ↓CM	< 0.0001 ↓SM
#22	P-B des1-12	0.8 ± 0.4 (6/6)	0.6 ± 0.6 (31/35)	1.0 ± 1.4 (46/48)	•	•
#23	Hst-1	1.1 ± 1.6 (4/6)	1.1 ± 1.5 (24/35)	2.9 ± 2.9 (34/48)	•	0.02 ↓SM
#24	Hst-1 0P	**(0/6)**	0.2 ± 0.3 (15/35)	0.4 ± 0.7 (23/48)	NA	•
#25	Hst-3	**0.01 ± 0.03 (1/6)**	0.3 ± 0.6 (12/35)	1.2 ± 1.7 (26/48)	NA	0.008 ↓SM
#26	Hst-3 1/25	**(0/6)**	0.04 ± 0.1 (6/35)	0.9 ± 1.2 (25/48)	NA	0.0001 ↓SM
#27	Hst-3 1/24	**(0/6)**	0.8 ± 1.7 (15/27)	3.0 ± 3.4 (40/48)	NA	< 0.0001 ↓SM
#28	Cyst. A	2.0 ± 1.4 (6/6)	1.2 ± 1.2 (35/35)	2.2 ± 1.9 (47/48)	•	0.001 ↓SM
#29	Cyst B (all proteoforms)	1.3 ± 1.1 (6/6)	1.2 ± 1.2 (34/35)	1.4 ± 1.3 (43/48)	•	•
#30	Cyst. C (all proteoforms)	**0.03 ± 0.07 (1/6)**	0.05 ± 0.2 (3/35)	0.6 ± 0.7 (32/48)	NA	< 0.0001 ↓SM
#31	Cyst. D-R_26_ des1-5	**0.4 ± 0.9 (1/6)**	0.5 ± 0.8 (19/35)	0.8 ± 1.1 (28/48)	NA	•
#32	Cyst. D-R_26_ des1-8	**(0/6)**	0.06 ± 0.1 (11/35)	0.1 ± 0.2 (15/48)	NA	•
#33	Cyst. S	**(0/6)**	0.3 ± 0.7 (11/35)	2.5 ± 0.5 (29/48)	NA	0.0007 ↓SM
#34	Cyst. S1 (all proteoforms)	11.3 ± 12.7 (6/6)	4.5 ± 3.8 (33/35)	10.3 ± 10.6 (45/48)	•	0.002 ↓SM
#35	Cyst. S2	1.9 ± 3.0 (2/6)	2.2 ± 3.6 (22/35)	3.8 ± 3.9 (45/48)	•	0.002 ↓SM
#36	Cyst. SN (all proteoforms)	10.2 ± 13.8 (4/6)	9.2 ± 11.1 (31/35)	20.7 ± 17.1 (47/48)	•	0.0005 ↓SM
#37	Cyst. SA	0.9 ± 1.9 (2/6)	0.8 ± 2.1 (9/35)	2.9 ± 3.8 (35/48)	•	<0.0001 ↓SM
#38	Tβ4	0.4 ± 0.3 (5/6)	0.4 ± 0.5 (24/35)	0.8 ± 1.0 (30/48)	•	•
#39	α-defensin 1	1.1 ± 0.7 (6/6)	1.4 ± 1.9 (33/35)	1.9 ± 2.9 (41/48)	•	•
#40	α-defensin 2	0.8 ± 0.6 (6/6)	0.7 ± 0.9 (31/35)	1.3 ± 1.7 (37/48)	•	•
#41	α-defensin 3	0.6 ± 0.7 (5/6)	0.3 ± 0.4 (21/35)	0.8 ± 1.3 (30/48)	•	•
#42	α-defensin 4	0.2 ± 0.1 (3/6)	0.1 ± 0.2 (15/35)	0.3 ± 0.4 (23/48)	•	•
#43	S100A9(S) + (S)ox	1.3 ± 1.5 (4/6)	1.5 ± 1.9 (24/35)	2.3 ± 2.7 (39/48)	•	•
#44	S100A9(S) 1P + (S)ox 1P	0.4 ± 0.5 (3/6)	0.6 ± 1.0 (16/35)	0.5 ± 1.0 (17/48)	•	•
#45	SLPI	0.2 ± 0.3 (3/6)	0.2 ± 0.8 (18/35)	0.1 ± 0.20 (10/48)	•	0.005 ↑SM
#46	S100A8 (all proteoforms)	**0.5 ± 1.1 (1/6)**	0.6 ± 1.1 (14/35)	0.2 ± 0.6 (9/48)	NA	0.02 ↑SM
#47	S100A8-SNO	**0.5 ± 1.1 (1/6)**	0.3 ± 0.7 (8/35)	0.1 ± 0.5 (6/48)	NA	•

a *p* values >0.05 are not statistically significant (•). In some cases, no statistical comparison was possible to carry out because of the absence of a protein in one or more groups (NA). Data are reported in bold when frequencies are <2 for group. The numbers in column 1 correspond to those ones reported in Figure 3 describing the volcano test results.

### Differentiation between Subgroups of SM Patients

**Figure 2:**
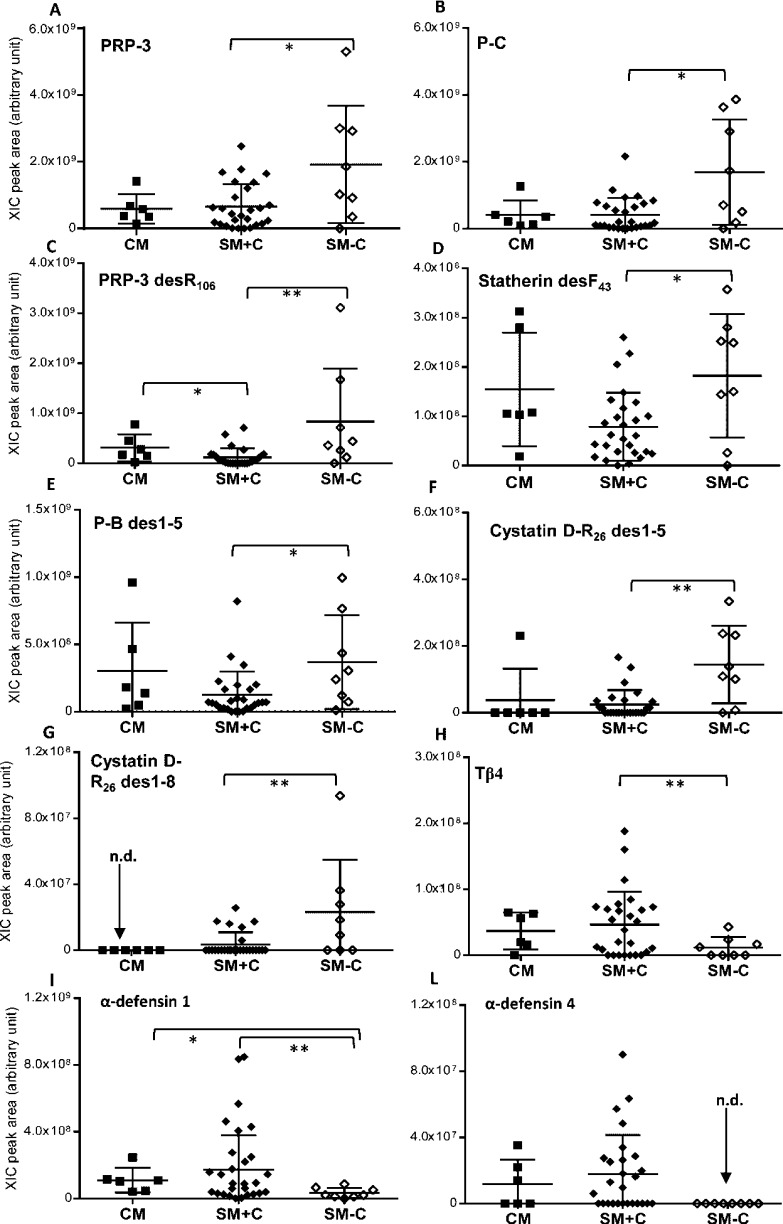
XIC peak area distribution of the proteins and peptides with levels statistically different between CM, SM+C, and SM-C patients. (A) PRP-3, (B) P-C peptide, (C) PRP-3 desR106, (D) statherin desF43, (E) P-B peptide des1-5, (F, G) cystatin D-R26 des1-5 and des1-8, and (H–L) Tβ4 and α-defensins 1 and 4. Statistical significance is indicated with asterisks (*p < 0.05; **p < 0.01). n.d. = not detected.

We compared the two subgroups of SM patients with and without cutaneous symptoms, called SM+C and SM-C, and identified some significant differences. PRP-3, PRP-3 desR_106_, P-C peptide, statherin desF_43_, and P-B des1-5 were more abundant in SM-C than in the SM+C subgroup (Figure 2A–E).

**Figure 3:**
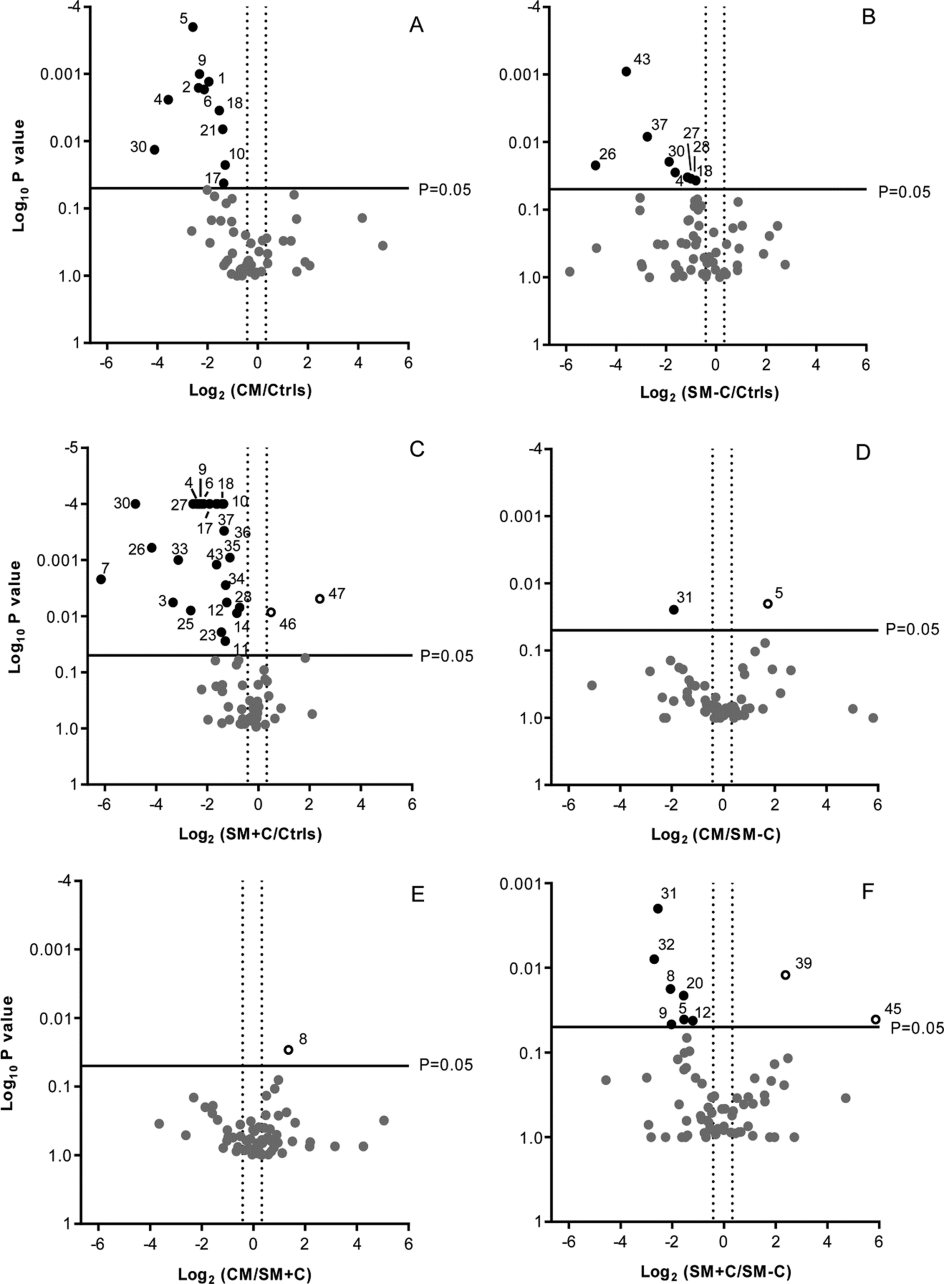
Volcano plots obtained by comparing the levels of proteins and peptides (A–C) in Ctrls versus CM, SM-C, and SM+C groups, (D, E) in CM versus SM-C and SM+C groups, and (F) in SM+C versus SM-C.

Moreover, both cystatin D-R_26_ des1-5 and des1-8 were more abundant in SM-C patients than in those with cutaneous symptoms, both SM+C and CM groups (Figure 2F,G). The levels of Tβ4 and α-defensins 1 and 4 were significantly less abundant in SM-C than in the SM+C group (Figure 2H–L). α-Defensin 4 has never been detected in samples from SM-C patients. The significantly high level of total S100A8 in SM patients was a consequence of the higher abundance of S100A8-SNO in the subgroup of the SM+C subgroup with respect to the Ctrls (*p* = 0.03, not shown in the Figure 3). This proteoform of S100A8 was detected in just 1 SM-C patient. Some of the differences highlighted by the statistical test based on two-group comparison, Mann–Whitney or Welch-corrected *t* tests, were confirmed by a volcano plot test (Figure 3).

In the plot, each number corresponds to the protein/peptide as listed in Table 3. The test evidenced different salivary protein profiles of the healthy controls and the groups of patients, CM, SM+C, and SM-C (Figure 3A–C). Higher levels of PRP-3 and lower levels of cystatin D-R_26_ des1-5 in CM patients differentiated them from the SM-C group (Figure 3,D), while higher levels of PRP-3 desR_106_ in CM patients were distinctive with respect to the SM+C patients (Figure 3,E). SM+C patients were differentiated from SM-C patients for the higher levels of α-defensin 1 and lower levels of PRP-3 and PRP-3 desR_106_, P-C peptide, statherin desF_43_, P-B peptide des1-5, and cystatin D-R_26_ des1-5 and des1-8.

### Correlation Analysis with Tryptase and with Oral Aphtosis and GI Symptoms

Except for 4 CM patients and 4 SM-C patients, all the others manifested GI symptoms, whereas aphtosis was detected in 13 SM patients (12 SM+C and 1 SM-C) and in 2 CM. There was no difference in the levels of investigated peptides/proteins by subgrouping the patients on the basis of the presence or absence of aphtosis. CM patients showed a normal tryptase concentration (6.3 ± 1.9 μg/L, mean ± standard deviation) compared to both groups of SM patients, where tryptase concentration was 28.3 ± 23.1 and 28.2 ± 19.1 μg/L in SM and SM + C, respectively. The XIC peak area of the proteins/peptides determined in each patient was correlated to the tryptase concentration measured in the same patient at the time of sample collection. The analysis performed by considering the entire SM group resulted in a significant positive correlation of statherin des1-10 and des1-13, P-B des1-4, and cystatin D-R_26_ des1-5 (Table 4). Different results were obtained by considering the two SM subgroups, SM+C and SM-C. Indeed, no correlation was obtained in the SM+C group, whereas in the SM-C group, the levels of monophosphorylated PRP-1 and PRP-3, PRP-3 desR_106_, statherin truncated forms, desD_1_, des1-10, and des1-13, all the four truncated forms of the P-B peptide, and cystatin D-R_26_ des1-5 correlated positively with tryptase concentration, while a negative correlation was found with the Tβ4 level. The correlation plots are reported in Figure S1.

**Table 4:** Results of Correlation Analysis between Salivary Proteins/Peptides and Tryptase Levels in SM Patients and in the Subgroup SM-C^a^

	SM (*n* = 35)		SM-C (*n* = 8)		
protein/peptide	*p* value	*R*	*p* value	*R*	distinctive between SM-C and SM+C
PRP-1 1P			0.04 (+)	*0.77*	
PRP-3 1P			0.03 (+)	*0.77*	
PRP-3 desR_106_			0.007 (+)	*0.88*	Y (↑ in SM-C)
Statherin desD1			0.01 (+)	*0.86*	
Statherin des1-10	0.03 (+)	*0.40*	0.02 (+)	*0.79*	
Statherin des1-13	0.02 (+)	*0.40*	0.02 (+)	*0.79*	
P-B des1-4	0.02 (+)	*0.38*	0.002 (+)	*0.93*	
P-B des1-5			0.02 (+)	*0.81*	Y (↑ in SM-C)
P-B des1-7			0.01 (+)	*0.86*	
P-B des1-12			0.006 (+)	*0.89*	
Cystatin D-R_26_ des1-5	0.02 (+)	*0.40*	0.005 (+)	*0.90*	Y (↑ in SM-C)
Thymosin β4			0.005 (−)	*–0.84*	Y (↓ in SM-C)

a In the table, the *p* value and the Spearman *R* coefficient are indicated. The significant difference between SM-C and SM+ C is indicated (Y).

## Discussion

The aim of this study was to investigate possible variations in the protein salivary profiles of patients affected by mastocytosis with respect to gender/age-matched healthy controls. We underline that the low number of patients in the group with just cutaneous mastocytosis, which is a rare disease subtype in adults, has often made it difficult to perform statistical analysis. Despite this limitation, the study highlighted important quantitative/qualitative differences between patients and controls and between the subgroups of patients. The salivary profiling of the patients showed significantly low abundance of aPRPs, statherin, P-B peptide, and histatins, proteins/peptides specific to the oral cavity and secreted by salivary glands. In the SM group, the levels of some naturally occurring fragments of aPRPs, statherin, and P-B peptide allowed us to distinguish the patients with only systemic symptoms, SM-C, from those with additional cutaneous symptoms, SM+C, which showed significantly lower levels of PRP-3 and PRP-3 desR_106_, P-C peptide, statherin desF_43_, also called SV1, and P-B peptide des1-5. Apart from the PRP-3 and P-C peptide, which originate from a pre-secretory proteolytic event occurring on the PRP-1 proteoform, the others probably originate from post-secretory proteolysis in the glandular ducts or in the oral cavity.^23^ The decreased levels of these salivary proteins and peptides could result in impaired homeostasis control of the oral cavity in mastocytosis patients; indeed, they are involved in the formation of the oral protein pellicles important to modulation of the colonization of microbial hosts and protection of the enamel and the mucosa epithelium.^38^ Histatin 1 plays an important role in the wound healing of the oral mucosa and in modulation of cell migration.^39^ Moreover, mastocytosis patients might show a frailer defense against oral infections because of the very low levels of histatin 3 and their truncated forms Hst-3 1/24 and 1/25, especially in CM patients. They exert powerful antibacterial and antifungal activities, belonging to the host defense antimicrobial peptides (AMPs), and thus are key components of the innate immune system.^41,42^ All the cystatins detectable in saliva, except cystatin B, showed a low abundance in the patients: S-type cystatins and cystatin A were less concentrated especially in SM patients, cystatin D-R_26_ were less concentrated in all the patients especially in CM patients, and cystatin C was almost absent. Cystatins are important inhibitors of endogenous and exogenous proteases and are involved in the inflammatory processes and in the innate immune response;^42^ S-type cystatins play this function in the oral cavity, suppressing some viral, bacterial, and fungal infections of the oral cavity by inhibiting exogenous cysteine proteinases.^43,44^ Cystatin C is able to modulate migration of monocytes and T cells.^45^ All the cystatins can regulate the cathepsin activity. Cystatin C inhibits lysosomal cathepsins B, H, K, L, and S.^42,46^ Cystatin D acts as an inhibitor of the cathepsins B, H, L, and S.^47^ Cystatin A is an inhibitor of cathepsins B, L, and H in the epidermis, lymphoid tissue, and oral mucosa, while S-type cystatins act as inhibitors of cathepsin C.^43,48^ The anti-cathepsin activity of SN is implicated in the destruction of periodontal tissues.^49^ It is necessary to underline that activated mast cells release cathepsins, in particular cathepsin C.^50^ It can be supposed that an impaired cathepsin regulation in mastocytosis patients might lead to a major inclination through oral inflammation and mucosal lesions, which are frequently associated with the disease.^20,21,51,52^ Moreover, because the salivary cystatins can be expressed in several tissues and secreted in other biological fluids,^43^ the condition evidenced in saliva might reflect a systemic state, where the altered suppression of cathepsin activity results in a disproportionate inflammatory and allergic responses. Interesting results have been found about thymosin β4 and α-defensins 1 and 4, when the SM patients were divided based on the presence of additional cutaneous symptoms. Indeed, the lowest levels of these components were measured in SM-C patients. Similar to histatin 3 and its fragments, α-defensins are antimicrobial peptides belonging to the AMPs,^40^ being implicated in host defense and homeostasis of tissues and biological fluids by recruiting immune cells in the infection site.^53^ α-Defensins can act both as antimicrobial peptides and as modulators of the inflammatory response through regulation of the cytokine production.^54^ Their decreased levels in SM-C patients, in addition to the downregulation of other salivary AMPs, could contribute to the weakening of the innate system defense of these subjects and contribute to higher local inflammation. Tβ4 stimulates cell growth and proliferation and acts as an antibacterial, anti-inflammatory, and antiapoptotic agent in saliva.^55^ A study performed on mast cells in vitro demonstrated that Tβ4-stimulated mast cells release mediators involved in angiogenesis and wound healing by a process that probably involved the actin-binding motif.^56^ An immunohistochemical study proposed Tβ4 as a novel marker of mast cells since it shows a degree of immunoreactivity comparable, in sensitivity, to chymase and tryptase.^57^ Indeed, it was demonstrated that mast cells infiltrating normal dermal and mucosal tissues, such as the tumoral ones, exhibit strong expression of Tβ4.^57^ Our study did not analyze the Tβ4 expression into mast cells but show a reduced concentration in saliva of patients with the SM-C subtype. Despite the low number of samples, these results were suggestive of a possible study to investigate a reduction of salivary Tβ4 as a marker for systemic mastocytosis subtypes.

SLPI and the total S100A8 exhibited an opposite trend with respect to the other proteins and peptides, being upregulated in SM patients, but not in CM. . SLPI is an anti-inflammatory and antimicrobial protein produced by neutrophils and macrophages associated with the respiratory tract mucosa and parotid and submandibular glands.^58^ SLPI acts as a serine protease inhibitor of cathepsin G, elastase, and trypsin released from many cell types and chymase and tryptase from mast cells.^59^ The result obtained on SLPI in this study was indicative of an anti-inflammatory mechanism linked to the excessive mast cell activity in our patients and that might compensate for the loss of cathepsin inhibitors observed in this study. S100A8 is considered as an ROS scavenger, being particularly sensitive to oxidative cross-linking and massive oxidation, and has acquired the capacity to reduce oxidative damage.^60^ The increased levels of S100A8 particularly as a nitrosylated proteoform in the subgroup of SM+C could suggest a protective event associated with heightened oxidative stress in mastocytosis.^61^ The result appeared remarkable by considering that S100A8-SNO can suppress mast cell-mediated inflammation by reducing leukocyte adhesion and extravasation, as demonstrated in a study performed in the rat mesenteric microcirculation.^62^ It was interesting to observe the strong correlation between serum tryptase and the levels of some salivary peptides/proteins especially in the subgroup of SM-C patients. Tryptase is a trypsin-like enzyme used as a marker of mast cell activity. Mast cells release tryptase also into saliva, where it was proposed as a diagnostic tool to test food allergies.^63^ Unbalanced mast cell release of proteases converts its role from protective to damaging. It would be interesting to evaluate how the release of mediators by activated mast cells in the oral cavity could be associated with the variation of the levels of specific salivary proteins/peptides. It was intriguing to have found that Tβ4, PRP-3 desR_106_, cystatin D-R_26_ des1-5, and P-B des1-5, which correlated with tryptase, allowed us to distinguish the two subgroups of SM patients. These results were suggestive of considering them as promising biomarkers for a differential diagnosis of the disease.

## Conclusions

The proteomics characterization of the acid-soluble fraction of intact salivary proteins revealed to be a useful approach to highlight significant variations in the protein profiles between healthy controls and mastocytosis patients and within disease subgroups. Differences associated with the pathological status were found in the levels of both proteins/peptides secreted by salivary glands and were specific to the oral cavity and proteins/peptides released from epithelial cells and leukocytes, which are expressed also in other tissues. Moreover, the possibility of distinguishing the two SM subtypes, with and without cutaneous lesions, based on the levels of specific salivary peptides/proteins and the correlation found between these components and tryptase showed to be encouraging in the biomarker discovery for the disease classification.

## Supporting Information Available

Figure S1: correlation plots of the tryptase concentration measured in the SM-C patients and the peptides/proteins listed in Table 4 (PDF)

## ABBREVIATIONS

CM: cutaneous mastocytosis
SM: systemic mastocytosis
SM+C: systemic mastocytosis with additional cutaneous lesions
SM-C: systemic mastocytosis without additional cutaneous lesions
Ctrls: healthy controls
aPRPs: acidic proline-rich proteins
Hst: histatin
Tβ4: thymosin β4
SLPI: antileukoproteinase
WHO: World Health Organization
RP-HPLC-ESI-IT-MS: reverse-phase high-performance liquid chromatography separation coupled to electrospray-ion trap mass spectrometry
PTMs: post-translational modifications
GI: gastrointestinal symptoms
TFA: trifluoroacetic acid
ACN: acetonitrile
FA: formic acid
TIC: total ion current
XIC: extracted ion current
CID: collision-induced dissociation
HCD: higher-energy collisional dissociation
AMPs: antimicrobial peptides
FDR: false discovery rate
