# Supplementary material for: Top-Down Proteomics of Human Saliva Discloses Significant Variations of the Protein Profile in Patients with Mastocytosis
Source: J Proteome Res. 2020 Jun 24;19(8):3238–53. doi: 10.1021/acs.jproteome.0c00207 (PMC8008451; doi:10.1021/acs.jproteome.0c00207)
Supplement: Supplementary file 1 — pr0c00207_si_001.pdf [file pr0c00207_si_001.pdf]

Top-down proteomics of human saliva discloses significant variations of the protein profile in patients with mastocytosis.

Simone Serrao[a]‡, Davide Firinu[b]‡, Alessandra Olianas[a]‡, Margherita Deidda[b], Cristina Contini[a], Federica Iavarone[c,d], M. Teresa Sanna[a], Mozhgan Boroumand[e], Francisco Amado[f], Massimo Castagnola[e], Irene Messana[g], Stefano Del Giacco[b]‡, Barbara Manconi[a], Tiziana Cabras[a]‡.*

[a]Dipartimento di Scienze della Vita e dell'Ambiente, Università di Cagliari, Cagliari, Italia.

[b]Dipartimento di Scienze Mediche e Sanità Pubblica, Università di Cagliari, Italia.

[c]Dipartimento di Scienze Biotecnologiche di Base, Cliniche Intensivologiche e Perioperatorie, Università Cattolica del Sacro Cuore, Roma, Italia, and [d]Fondazione Policlinico Universitario A. Gemelli IRCCS, Roma, Italia. [e]Laboratorio di Proteomica e Metabonomica-IRCCS Fondazione Santa Lucia, Roma. [f]QOPNA, Mass spectrometry center, Department of Chemistry, University of Aveiro, Portugal.

[g]Istituto di Scienze e Tecnologie Chimiche “Giulio Natta”, Consiglio Nazionale delle Ricerche, Roma, Italia.

*** Corresponding Author:** bmanconi@unica.it

‡These authors contributed equally.

Figure S1. Correlation plots of Tryptase concentration measured in the SM-C patients and the peptides/proteins listed in Table 4.

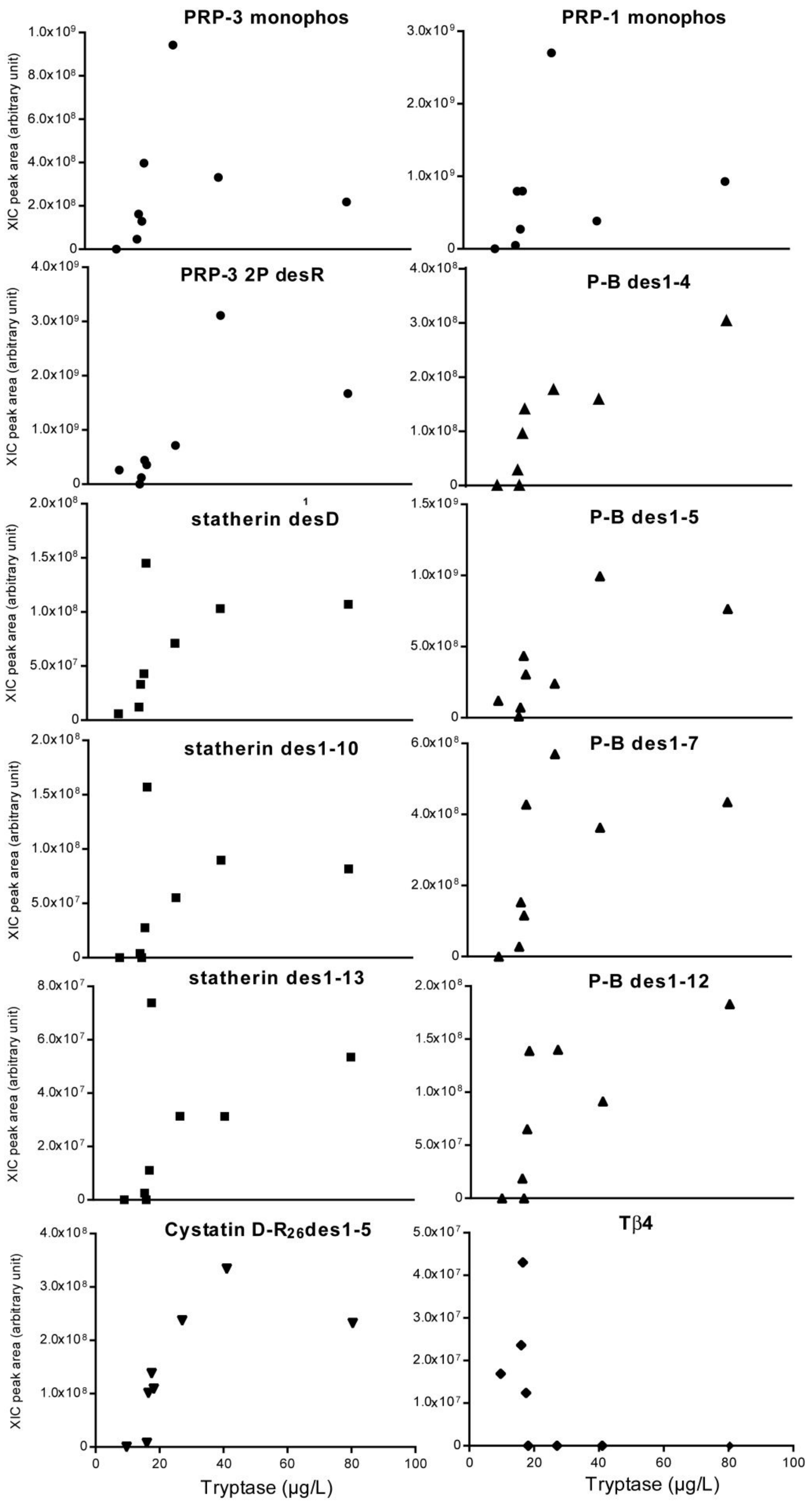
PRP-3 monophos
PRP-1 monophos
PRP-3 2P desR
P-B des1-4
statherin desD
P-B des1-5
statherin des1-10
P-B des1-7
statherin des1-13
P-B des1-12
Cystatin D-R26des1-5
Tβ4
XIC peak area (arbitrary unit)
Tryptase (µg/L)
0
20
40
60
80
100
